# Extracellular to Intracellular Body Water and Cognitive Function among Healthy Older and Younger Adults

**DOI:** 10.3390/jfmk7010018

**Published:** 2022-02-05

**Authors:** Jinhyun Lee, Richard K. Shields

**Affiliations:** Department of Physical Therapy and Rehabilitation Science, Roy J. and Lucille A. Carver College of Medicine, The University of Iowa, Iowa City, IA 52242, USA; jinhyun-lee@uiowa.edu

**Keywords:** cognitive function, body composition, aging, blood–brain barrier, inflammation

## Abstract

Compromised cognitive function is associated with increased mortality and increased healthcare costs. Physical characteristics including height, weight, body mass index, sex, and fat mass are often associated with cognitive function. Extracellular to intracellular body water ratio offers an additional anthropometric measurement that has received recent attention because of its association with systemic inflammation, hypertension, and blood–brain barrier permeability. The purposes of this study were to determine whether extracellular to intracellular body water ratios are different between younger and older people and whether they are associated with cognitive function, including executive function and attention, working memory, and information processing speed. A total of 118 healthy people (39 older; 79 younger) participated in this study. We discovered that extracellular to intracellular body water ratio increased with age, was predictive of an older person’s ability to inhibit information and stay attentive to a desired task (Flanker test; R^2^ = 0.24; *p* < 0.001), and had strong sensitivity (83%) and specificity (91%) to detect a lower executive function score. These findings support that extracellular to intracellular body water ratio offers predictive capabilities of cognitive function, even in a healthy group of elderly people.

## 1. Introduction

There is an estimated two-fold increase in healthcare costs associated with impaired cognitive function among older adults in the United States [1]. Fluid cognition is one type of cognitive function that is known to decline with age [2], adversely impact quality of life [3], and be associated with changes in the hippocampus of the brain [4]. Fluid cognition includes executive function, attention, working memory, and information processing speed—all factors that impact the health of older people and that are measurable using the National Institutes of Health (NIH) Cognitive Toolbox [5].

Anthropometric factors such as age, percentage body fat, lean muscle mass, and body mass index (BMI) are broadly associated with cognitive function decline [6]. Body water compartment levels offer an additional anthropometric measurement that has received recent attention [7]. Total human body water consists of approximately 40 percent extracellular water (blood plasma and interstitial) and approximately 60% intracellular water (within cells)—both of which are measurable using contemporary bioimpedance techniques [7]. 

Several links between body water compartment volumes and pathology may con-tribute to impaired cognitive function [8,9,10,11]. Increased edema and systemic inflammation [9,11], impaired brain blood flow [8,9,10], impaired brain metabolism [8,9,10], and impaired systemic renal function [11] are examples of pathologies associated with changes in body water volume and may be associated with impaired cognitive function. During healthy aging there are subtle changes in edema/inflammation [12,13], blood flow [14], metabolism [15], muscle atrophy [16], and renal function [17] that may be associated with body water volume. Therefore, we sought to assess whether body water volumes explain variation in cognitive function beyond that associated with age, height, weight, percentage body fat, sex, and lean/fat mass in healthy younger and older people.

We identified two reports that related body water compartment levels to cognitive function in humans [18,19]. The first showed significant increases in extracellular water, edema, and cognitive function in people with more severe long-term diabetes [19]. The second study showed no significant association between cognitive function and extracellular water in elderly people in a nursing home [18]. To the best of our knowledge, no study has assessed whether extracellular/intracellular water ratios are associated with cognitive function in healthy younger and older people. Importantly, there is a lack of knowledge as to whether extracellular/intracellular water is more aligned with certain types of cognitive function, such as executive function, attention, working memory, or information processing speed as measured with the NIH cognitive function toolbox [5]. Our long-term goal is to understand whether physical characteristics of healthy older people can identify those at risk for mild to moderate cognitive impairment as related to executive function, attention, working memory, or information processing speed. 

Measurement of body water compartment levels in humans using bioimpedance technology has enjoyed many technological advancements [7]. By measuring the resistance to small currents injected into the body, the fat, muscle, bone, intracellular water, and extracellular water may be estimated [7]. 

The purposes of this study were to determine whether body water percentages are different between younger and older people and whether extracellular and intracellular body water ratios are associated with various types of fluid cognition, including executive function and attention, working memory, and information processing speed. The extent to which the extracellular to intracellular water ratio explains variance in cognitive function relative to age, height, weight, BMI, lean/fat mass, sex, and percentage body fat was examined. We hypothesized that the extracellular to intracellular water ratio would be an important addition to the anthropometric predictors of cognitive function in both younger and older healthy people. 

## 2. Materials and Methods

One hundred and eighteen healthy people participated in this study (Table 1). 

We included people who were between 18 and 80 years old who had a minimum of a high school education, could read English, and could comprehend verbal communications. We excluded people if they had a known neurological condition that affected upper and lower extremity movement, had been diagnosed dementia, had a known neurocognitive disorder, or had a pacemaker for cardiovascular pathology. All participants who began the study completed the study. One participant was removed because of technical problems with data collection. 

We dichotomized our data set into an “older” group and a “younger” group because there was a natural break point in the data for those who were greater than 48 years old (mean 62 years +/−6) and those who were less than 39 years old (mean 25 years +/−4). However, because we know that chronological age is not always associated with biological age, we assessed the entire group using age as a continuous variable. The relative homogeneity of each group (sd < 6 years) supports that our age groups were distinctly different. 

The institutional review board of the University of Iowa approved this study, in accordance with the Declaration of Helsinki. All participants signed an informed consent prior to participating in the study. 

### 2.1. Cognitive Testing

We used the National Institutes of Health (NIH) toolbox, a reliable and rigorous measurement of cognitive function [20,21,22,23,24], on an iPad (tablet) to assess the following five subtests in order: the Pattern Comparison Processing Speed Test (Pattern Comparison), the Picture Sequence Memory Test (Picture Sequence), the Dimensional Change Card Sort Test (Dimensional Change), the Flanker Inhibitory Control and Attention Test (Flanker), and the List Sorting Working Memory Test (List Sorting)—all tests that focus on executive function [20,21,22,23,24]. All tests were designed for individuals aged 3 to 85 years [22,24,25,26] except for the list sorting test (7 to 85 years) [27]. Generally, all tests were administered between 2:00 and 3:00 p.m., approximately 2 h after lunch. To meet our participants schedule, minor adjustments in the afternoon time were made occasionally. Participants did not eat or drink fluids during the cognitive testing. All participants were encouraged to refrain from exercise the day of the testing and to refrain from alcohol 24 h before testing. All participants were asked to use the restroom ~15 min before testing. The specific administration instructions have been described in a previous report [28,29]. 

The Pattern Comparison Processing Speed Test is designed to test processing speed [24]. Participants were shown two pictures, and if they looked the same, they were asked to choose yes [24]. The time was used to score a participant’s processing speed. 

The Picture Sequence Memory Test is designed to test episodic memory [30]. Participants were shown multiple images and were asked to remember the order of the images [31]. The number of images that could be accurately retained constituted a participant’s episodic memory.

The Dimensional Change Card Sort Test is designed to test executive function [20]. Participants were given three pictures and instructed to utilize the first picture as a reference and then asked to select one of the two subsequent pictures based on the criterion specified by the iPad application (color or shape) [25]. For example, participants were shown a reference picture of a red tiger. The application then prompted them to select one of the two subsequent pictures (a red car and a green tiger) based on the color of the reference picture [25]. The speed and accuracy of this task were used to characterize executive function. 

The Flanker Inhibitory Control and Attention Test (Flanker) is designed to test attention and executive function [20]. Participants were shown five different arrows that pointed left or right and were asked to focus only on the middle arrow and choose, from a pair of arrows, which one was like the middle arrow [26]. The speed of completing this task was used to assess test attention and executive function.

The List Sorting Working Memory Test is designed to test working memory [21]. Participants were shown multiple objects in order and then asked to recall all objects from smallest to biggest [28]; these objects were organized into one category (animals), and then two categories (food and animals) [28]. The number of accurate objects recalled was used to assess working memory.

The average test completion time was ~30 min for all 5 tests [32]. Tests were performed in the order presented, and there were no rests between each cognitive test.

### 2.2. Regional Bioimpedance Analysis

A regional bioimpedance analysis device (InBody S10, InBody, Seoul, Korea) was used to provide regional anthropometric measurements. The device utilizes six frequencies, including three frequencies below 100 kHz (1, 5, 50) and three frequencies above 100 kHz (250, 500, 1000). The regional bioimpedance analysis was completed before the cognitive function testing (~2:00 p.m.) and ~2 h after eating. Participants did not eat or drink fluids during the test session. As indicated above, all participants were encouraged to refrain from exercise the day of the testing and to refrain from alcohol 24 h before testing. All participants were asked to use the restroom ~15 min before testing began. All impedance assessments were completed in the seated position after an acclimation time of 15 min as per manufacturer recommendations. Participants were instructed to maintain 80° knee flexion, have bare feet, keep shoulder width distance between limbs, and maintain trunk and arm separation by 15°. Participants were asked to not slouch, talk, or move and to not have contact with any metal (e.g., jewelry) during the measurements [33]. Eight electrodes were applied to four different regions of both left and right limbs (two electrodes for one region): left and right legs and left and right hands. Following the seated posture acclimation, the test duration was less than two minutes per participant [33]. 

The InBody device provides anthropometric indices, including total body water, intracellular water, and extracellular water content. Intraclass correlation coefficients of repeated tests within our lab were greater than 0.91 for both intracellular water and extracellular water. These bioimpedance measures were validated to the gold standard, which uses sodium bromide to trace extracellular water (R^2^ value of 0.94) [34]. 

### 2.3. Statistical Analysis

Sample size for this study was calculated using the estimated variance from a previous report [19], 80% power, and the G * Power software (version 3.1.9.2, Dusseldorf, Germany). To assess the hypotheses, parametric (independent t-test) and non-parametric statistical tests (Mann–Whitney rank sum test) were conducted to assess cognition and body water compartment differences between the younger and the older adults. (Statistical significance was assigned at *p* ≤ 0.05). Non-parametric tests, including the Kolmogorov–Smirnov and Shapiro–Wilk were used when/if normality and homogeneity of variance assumptions were violated. Univariate regression analysis was conducted to identify associations among anthropometric data and NIH Toolbox cognition data. Coefficients of determination were used to determine the strength or weakness of the predictive model. Stepwise analysis was used to determine the best set of variables to predict cognitive function (F to enter = 1.6, F to remove = 1.5). 

## 3. Results

### 3.1. Fluid Cognition among Young and Older Groups

Table 1 shows that height, weight, body mass index, percentage (%) body fat, lean/fat mass, and age were different for young versus the older group. Each NIH Toolbox test for fluid cognition showed less cognitive function for the older group as compared with the younger group, with effect sizes of 2.16, 1.11, 1.43, 1.75, and 0.66 for Pattern Comparison, Picture Sequence, Dimensional Change, Flanker, and List Sorting, respectively. Specifically, the fluid cognition was higher for the younger group as compared with the older group for the overall composite score (U = 928.5, *p* < 0.01), Dimensional Change (U = 540; *p* < 0.01), Picture Sequence (U = 689.5, *p* < 0.01), List Sorting 5.4 (t = 3.22, *p* < 0.01), Pattern Comparison (t = 10.78, *p* < 0.01), and Flanker (t = 8.69, *p* < 0.01) tests. Despite an overall healthy cohort, we found significant changes in cognitive function in the older group as compared with the younger group. 

### 3.2. Associations with the Type of Cognitive Function Assessed

Table 2 shows the correlational analysis for group characteristics for each of the NIH Toolbox fluid cognition tests for the combined, the young, and the older groups. 

Age explained 24, 30, 42, and 51% of the variance in the cognitive assessment score for Picture Sequence, Dimensional Change Card Sort, Flanker, and Pattern Comparison, respectively, for the combined group of participants. Age explained less than 8% of the variance of the List Sorting cognitive test scores. Extracellular/intracellular water ratio explained 11, 24, 38, and 32% of the variance in cognitive scores for Picture Sequence, Dimensional Card Change Card Sort, Flanker, and Pattern Comparison, respectively, for the combined group. Extracellular/intracellular water ratio only explained 6% of the variance of the List Sort cognitive test scores. Neither age nor extracellular/intracellular ratio explained significant variance among the five cognitive test scores for the younger or older groups, with one exception. The extracellular/intracellular ratio explained 24% of the variance of the Flanker test scores for the older group while age explained 5%. Because of this finding, we focused on the Flanker Inhibitory Control and Attention Test (prime features of executive function) for a more in-depth analysis. 

### 3.3. Extracellular/Intracellular Water and the Flanker Test

**Figure 1:**
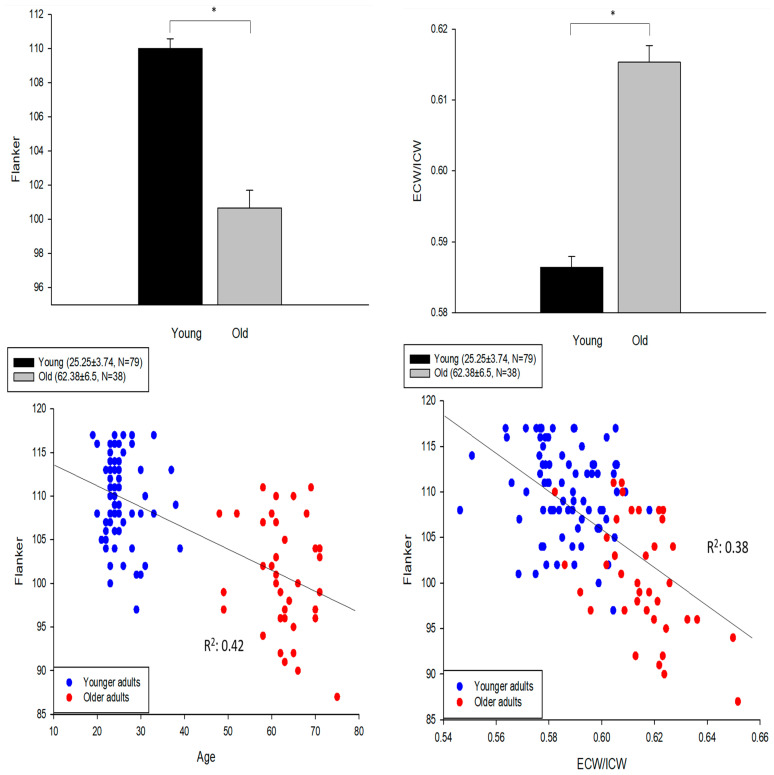
Upper left: Younger adults (*n* = 79) had higher Flanker test scores as compared with the older adults (t = 8.69, df = 115, and * *p* < 0.01; error bar: SE). Upper right: Younger adults had lower extracellular to intracellular water ratio (ECW/ICW) than the older adults (t = −10.71, df = 115, * *p* < 0.01). Lower left: A significant association between age and Flanker test (R^2^ = 0.42; *p* < 0.01). Lower right: A significant association between ECW/ICW and Flanker test (R^2^ = 0.38; *p* < 0.01).

Figure 1 upper panels show the magnitude of the difference between the younger and older groups for the Flanker test scores and the extracellular/intracellular body water ratio with effect sizes of 1.75 and 1.74, respectively. 

The lower left panel shows that age was correlated with the Flanker test scores (R^2^ = 0.42; *p* < 0.01), although, as expected, the data were clustered based on the age distribution of our sample of convenience. The Flanker scores, normalized to age, were not normally distributed (Kolmogorov–Smirnov; *p* < 0.05). The lower right panel shows that when age was replaced by the extracellular/intracellular body water ratio of each participant, there was a moderate correlation with the Flanker test scores (R^2^ = 0.38; *p* < 0.01). The Flanker test scores, when normalized to the body water ratios, were normally distributed (Kolmogorov–Smirnov; *p* > 0.05). Age was also correlated with extracellular/intracellular water (R^2^ = 0.45; *p* < 0.01). 

**Figure 2:**
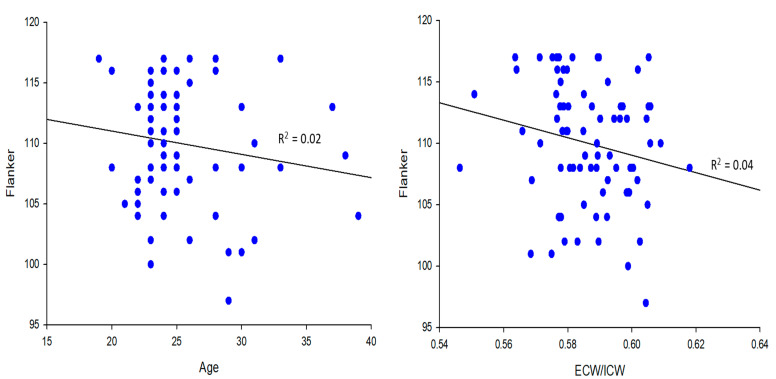

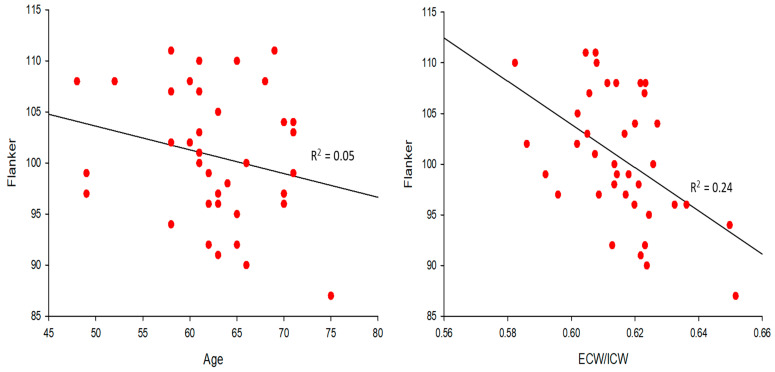
Upper and lower left panels: Age was not associated with the Flanker test scores in the older group (R^2^ < 0.05; *p* > 0.05). Upper and lower right panels: There was no association between extracellular/intracellular ratio and Flanker in the younger group, but there was an association between extracellular/intracellular ratio (ECW/ICW) and Flanker in the older group (R^2^ = 0.24; *p* ≤ 0.01).

The Figure 2 upper panels show that there was no correlation between age and the Flanker test scores (R^2^ = 0.02; *p* > 0.05) and between the ratio of extracellular/intracellular water and the Flanker scores in the younger group (R^2^ = 0.04; *p* > 0.05). 

Age was also not correlated with the ratio of extracellular/intracellular water in the younger group (*p* > 0.05, R^2^ = 0.03). The lower panels show that there was no correlation between age and the Flanker test scores (*p* > 0.05, R^2^ = 0.05) in the older group. However, the ratio of extracellular/intracellular water was significantly correlated with the Flanker test scores (*p* < 0.01, R^2^ = 0.24) in the older group. Age was not correlated with the ratio of extracellular/intracellular water in the older group (*p* > 0.05, R^2^ = 0.01). 

**Figure 3:**
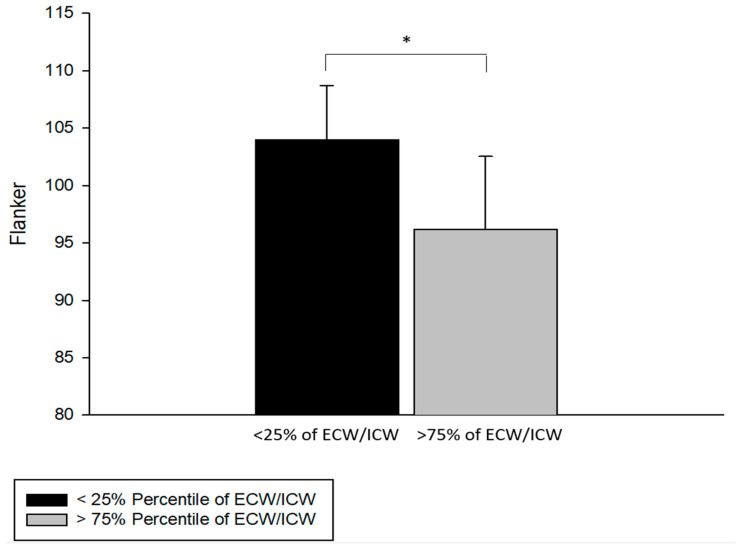
Less than 25% of extracellular/intracellular ratio (ECW/ICW) in older participants had higher Flanker test scores as compared with more than 75% of ECW/ICW in older participants (t = 3.013, df = 17, and * *p* < 0.01).

Using a cutoff score of 95 (mean for cohort was = 100) on the Flanker test as an arbitrary indicator of healthy people with lower executive function, the extracellular/intracellular water ratio had a sensitivity of 83% and a specificity of 91%. The Flanker test scores were significantly lower among those who had the highest extracellular/intracellular ratios (>75th percentile) as compared with those with the lowest ratios (<25th percentile; *p* < 0.005) among a healthy group of older people without chronic disease (Figure 3). 

**Figure 4:**
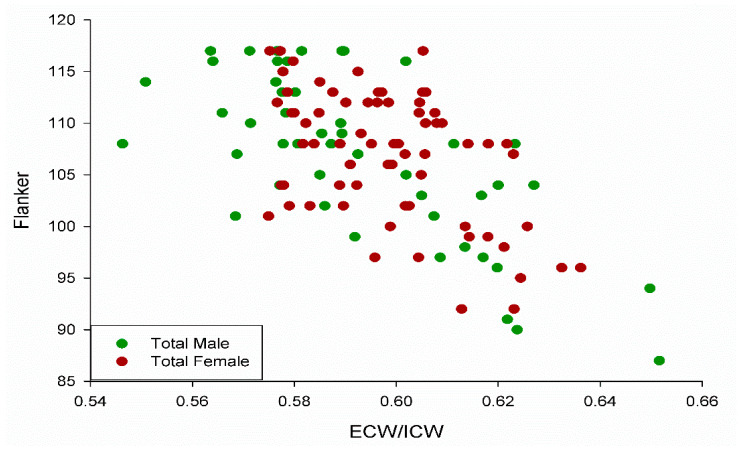
Scatterplot depicting the similar distribution between males and females in the study with R^2^ = 0.38, 0.51, and 0.25 for the total group, male group, and female group, respectively (all *p* < 0.05), for Flanker scores relative to extracellular/intracellular ratio (ECW/ICW).

The distribution of extracellular/intracellular ratios and Flanker scores were similar for male and female participants in the study (Figure 4). There was no difference between male and female participants for the Flanker test (U = 1598, *p* = 0.71).

In the male group, over 50% of the variance in the Flanker test was explained by the extracellular/intracellular water ratio; while 25% of the variance was similarly explained in the female group (all significant at *p* < 0.05).

### 3.4. Stepwise Regression Analysis to Predict Cognitive Function

Using each variable from Table 3 (sex, age, height, weight, BMI, percentage BF, lean/fat, sex, and extracellular/intracellular ratio) in a stepwise logistical model to predict executive function (Flanker), only two variables—age and extracellular/intracellular water ratio—were selected into the model.

The best equation to predict executive function in the combined young and older group was:

$$Flanker=184.006-\left(0.162\times \mathrm{Age}\right)-\left(119.104\times \frac{\mathrm{extracellular}}{\mathrm{intracellular}}\;ratio\right)\;\mathrm{with}\;a\;\mathrm{total}\;\mathrm{multiple}\;R^{2}=0.48$$

When predicting executive function among the older group (48–75 years of age), the extracellular/intracellular water ratio was the only variable that entered the model, yielding an R^2^ = 0.22. The best equation to predict executive function in the older group was:

$$Flanker=231.837-\left(213.187\times \frac{\mathrm{extracellular}}{\mathrm{intracellular}}\;ratio\right)$$

When predicting executive function among the younger group (19–39 years of age), percentage (%) body fat, BMI, age, and lean/fat ratio were selected into the model. The best equation to predict executive function in the young group was:

$$Flanker=116.324+\left(0.412\times \mathrm{BMI}\right)-\left(0.254\times \mathrm{Age}\right)-\left(1.530\times \frac{\mathrm{Lean}}{\mathrm{Fat}}\right)-\left(0.364\times \%\mathrm{body}\;\mathrm{fat}\right)\;\;\mathrm{with}\;a\;\mathrm{total}\;\mathrm{multiple}\;R^{2}=0.16$$

## 4. Discussion

We discovered that extracellular/intracellular body water ratios are significantly changed with age and that extracellular/intracellular body water ratios are predictors of cognitive function in a combined group of healthy younger and older participants. The extracellular/intracellular water ratio also emerged as the primary predictor of cognitive performance (Flanker test) within the older group. The extracellular/intracellular water ratio is most associated with a person’s cognitive ability to inhibit information and stay attentive to a desired task (as measured by the Flanker test). Taken together, these findings support that extracellular/intracellular body water ratio offers additional predictive capabilities of cognitive function, even in a healthy group of elderly people. 

A study assessing extracellular/intracellular water ratio and cognition in healthy older adults has not previously been undertaken. Only two studies have examined these parameters in nursing home clients [18] and among people with diabetes [19]. There was no correlation between extracellular body water and cognition among nursing home participants [18], a finding that is not congruent with our study. Our study included a larger sample size, younger and healthy participants, use of five domains within the NIH Toolbox for cognitive assessment, and a greater range of cognitive scores, all factors that likely contribute to this discrepancy [18].

Our findings are congruent with Low and colleagues, who discovered increased extracellular water was associated with decreased cognitive function in the attention and memory domains among people with various levels of diabetes [19]. A careful review of their results revealed that extracellular water, as an index of edema, was also aligned with pulse pressure, hypertension, and glycemic control [19]. The novelty of our study is that we detected that the Flanker test for executive function was significantly lower among those who had the highest extracellular/intracellular ratios (>75th percentile; *p* < 0.005) among a healthy group of older people without chronic disease. The range of scores for the Flanker test in our study was 87 to 117 (Mean = 100). If we classify all older people who scored 95 or lower on the Flanker test as having “lower cognitive function”, then the extracellular/intracellular ratio had an 83 percent probability of detecting those with lower cognitive performance (low false negative or sensitivity); while the extracellular/intracellular ratio had a 92 percent probability that those without “lower cognitive function” would not be selected (low false positive or specificity). This finding, coupled with more extensive longitudinal research, may provide the basis for developing a model that is sensitive to detecting healthy older people at risk for developing cognitive impairment. 

It is noteworthy that the Flanker test, which assesses executive function and attention, yielded the greatest correlation to the water compartment ratios as compared with several other fluid cognitive assessments. Mooijaart and colleagues found an association between attention/executive function and systemic inflammatory biomarkers, but they did not find an association between working or episodic memory and inflammation [35]. Li and colleagues reported that patients diagnosed with mild vascular cognitive impairment showed diminished information processing speed, attention, executive function, spatial function, and language function but no measurable change in memory [36]. Hence, it may be that our healthy group of older people with the highest extracellular/intracellular water ratios, who performed poorer on the Flanker test of executive function and attention, are demonstrating early changes in cognitive performance. The thread of evidence in the literature suggesting that attention and executive function show early signs of impaired cognitive change is an intriguing hypothesis and one that will require more extensive evaluation in the future.

The physical characteristics of the participants in our study, other than water content, were generally not predictive of cognitive test performance. More than likely, this was because we had a homogenous group of healthy younger and older participants with a relatively low variance in the physical characteristic measurements. Others have found significant correlations between BMI, percentage body fat, and lean/fat mass ratios and cognitive function [6,37,38]. The historic relationship among physical characteristics, metabolic syndrome, and cognitive function is well established; [6,38] albeit there are controversies about fat as being negatively associated with cognitive function in older males but positively associated with cognitive function in older females [38,39]. Regardless, among the older group there was no effect of sex on cognitive scores. 

As we search for more sensitive biomarkers to predict those at risk for developing mild cognitive impairment, we need to understand the variance within the healthy aging population. This thought is well-aligned with the notion that the healthy older people in this study still showed a significant decline in cognitive function as compared with younger people. Thus, the loss of cognitive function is often considered a natural path to healthy aging, but some go on to develop accelerated cognitive impairment and even Alzheimer’s Disease between 60 to 70 years of age [40]. An early detection program involving measurable physical characteristics, such as extracellular water, offers the opportunity to assess the extent to which cognitive status changes with lifestyle interventions that lower the extracellular to intracellular water balance ratio. Understanding whether diet, sleep, exercise, socioeconomic stress, and circadian metabolic control are part of the exposure that epigenetically tag genetic pathways associated with body water regulation warrants future investigation [41,42,43]. 

### 4.1. Potential Mechanisms Relating Extracellular Water and Cognitive Function

Pathology to the vascular system appears to be a primary path to impaired cognitive function. Leakage of blood plasma through the blood–brain barrier is believed to contribute to even mild cognitive impairment [36]. This leakage through the blood–brain barrier is measurable through imaging of the hippocampus of the brain and is tightly coupled with cognitive status [36]. Generally, blood plasma constitutes ~20% of all extracellular fluid in the body. When the blood plasma volume exceeds its normal level, it may contribute to hypertension [44], increased pulse pressure, and increased interstitial fluid edema [19]. It is well established that hypertension may damage blood vessels, leading to vessel stiffness and hypoperfusion of brain tissues (hippocampus), with permanent changes to the blood–brain barrier [36]. This altered blood flow may impair nutrients to the brain leading to hypometabolism of brain tissue. While the exact mechanism remains elusive, the blood–brain barrier leakage rate is known to be correlated with changes in hippocampal brain images, which are correlated with cognitive function scores in humans with known pathology [36]. Regrettably these studies are largely cross-sectional rather than longitudinal. Hence, a “cause and effect” relationship has not been established, but it is an important avenue to explore in future research.

Because over 20 percent of the extracellular fluid in the human body is blood plasma, our extracellular fluid measurements may provide a glimpse into early increases in blood plasma volume, which may be associated with hypertension and blood–brain barrier alterations in the brain [36]. A limitation in our work is that we did not assess blood pressure or quantify the activity level of our participants. All participants reported to have an active lifestyle with no diagnoses of any chronic diseases; however, hypertension is a silent pathology, and without quantified metrics on activity level, we cannot rule out that those who scored lower on the Flanker test may have had undetected hypertension and been less active than those who scored higher on the Flanker test. 

Our extracellular/intracellular water measurements were significantly higher in those who scored in the top 75th percentile on the Flanker test when compared with those who scored in the lowest 25th percentile. The extracellular/intracellular water ratio, as a predictor of healthy elderly with lower cognitive function, appeared to provide information that was not readily obtained by using BMI, percentage body fat, and lean to fat mass. 

The denominator of the body water ratio represents intracellular water. Intracellular water comprises over 50 percent of the total water in the human body. Seventy-five percent of skeletal muscle is intracellular water, and therefore, our findings have important implications to the field of gerontology, exercise physiology, skeletal muscle training, and rehabilitation. Muscle mass declines with age and is an important predictor of mortality [45], likely, as we lose mobility, we become more at risk for the development of several chronic diseases, including impaired cognitive function [46]. Intracellular water levels in the human body have been reported to explain some of the variance in muscle mass and function among a group of elderly people [7]. In our study, the intracellular water was significantly higher in those participants who scored in the top 75th percentile on the Flanker test when compared with those who scored in the lowest 25th percentile. We did not assess muscle size, function, or activity level; thus, we must be cautious about over interpreting the impact of intracellular water on skeletal muscle alone. This is fertile ground for future longitudinal investigations to ascertain whether changes in skeletal muscle size, strength, and activity levels are associated with changes in extracellular/intracellular water and cognitive function. 

### 4.2. Acute Regulation of Body Water Compartments

Our work raises several questions about acute changes in body water compartment ratios—either through hormonal factors or profuse sweating and dehydration. For example, a woman’s body water compartment levels fluctuate during the menstrual cycle, with the highest extracellular fluid content corresponding to the follicular phase when estrogen is high [47] and approximately 8% decline in extracellular water during the pre-ovulatory and luteal phase when estrogen is low [48,49]. Recent studies suggest an increase in working memory, reaction time, and abstract reasoning when estrogen levels are high [50,51]. Blood–brain barrier permeability appears to also change during various stages of the menstrual cycle [52], but the relationship between blood–brain barrier, hormonal levels, and cognitive function is not well established. A limitation of our study is that we did not quantify hormone levels for the younger or older woman in our study. However, close examination of our data reveals no difference in any cognitive test scores between male and female participants. Importantly, the extracellular/intracellular water ratio that emerged as a predictor of executive function for the older group was consistent between older males and older females (R^2^ = 0.24 and 0.25 for men and woman, respectively). This finding supports that the extracellular/intracellular ratio remained a strong predictor of cognitive function regardless of the sex of the older group.

Severe and prolonged dehydration is also well known to decrease cognitive function [53] and suggests that lower extracellular water may have a critical threshold. This also highlights a limitation of the body water compartment ratios, as the total body water may be reduced (dehydration) while the ratio may remain the same. We are currently exploring new methods to obtain an absolute value for total water content, adjusted for several anthropometric factors.

## 5. Limitations

This study offers new information about extracellular/intracellular ratios that explain variance in cognitive function among older people. However, there are several limitations discussed above and that warrant summarization. Specifically, we did not quantify participant activity levels, blood pressure, hormonal levels, hydration status, dietary habits, and skeletal muscle force generating capabilities, all of which would help advance our understanding of the relationship between cognitive function and extracellular and intracellular water balance. Although there appears to be sound physiological grounding for why older people with higher extracellular to intracellular body water ratios would score lower on cognitive function tests, much more work is needed in this field. Future investigations must build upon these findings to enhance our ability to identify those healthy elderly who are at risk of developing mild to moderate cognitive impairment. 

## 6. Conclusions

Extracellular/intracellular body water ratios explained significant variance in the attention and executive function performance in healthy elderly participants. The extracellular/intracellular body water ratio offers additional predictive capability of executive function beyond that of standard physical characteristics among the elderly. This study supports the need for future longitudinal trials to establish whether body water compartment analysis can identify those at early risk for developing impaired cognitive function and whether these measurements are sensitive to preventive lifestyle interventions.

## Figures and Tables

**Table 1:** Average age, weight, height, BMI, lean, fat, lean to fat ratio, percentage (%) body fat, extracellular/intracellular ratio (ECW/ICW), and all cognitive testing scores within the NIH Fluid Cognition Toolbox for the older and younger groups in the study. All variables except weight and lean were significantly different between young and older groups.

	Older (*n* = 39, 48–75 y, 20F)	Younger (*n* = 79, 19–39 y, 48F)	*p*-Value
Age	62.38 ± 6.5	25.25 ± 3.74	<0.05
Weight	78.41 ± 18.44 (kg)	72.1 ± 14.1 (kg)	=0.09
Height	167.77 ± 8.71 (cm)	172.91 ± 9.73 (cm)	<0.05
Body Mass Index (BMI)	27.8 ± 5.9	24.08 ± 4.3	<0.05
ECW/ICW	0.61 ± 0.02	0.59 ± 0.01	<0.05
Lean (kg)	14.35 ± 3.03	15.41 ± 3.41	=0.16
Fat (kg)	24.26 ± 12.74	15.15 ± 9.89	<0.05
Lean/Fat	0.93 ± 1.25	1.51 ± 1.14	<0.05
% Body Fat	29.84 ± 11.21	20.65 ± 10.5	<0.05
Pattern Comparison	101.56 ± 17.79	133.46 ± 13.22	<0.05
Picture Sequence	111 ± 14.19	124.3 ± 11.8	<0.05
Dim Change Card Sort	107.62 ± 7.21	115.84 ± 4.97	<0.05
Flanker	100.54 ± 6.3	110 ± 4.97	<0.05
List Sorting	107.44 ± 8.14	113.11 ± 9.59	<0.05

**Table 2:** Correlational analyses (R-squared values; bolded = *p* < 0.05) for multiple variables (weight, height, BMI, percentage body fat, lean mass to fat mass ratio, age, and extracellular/intracellular ratio (ECW/ICW) and each cognitive test within the combined, older, and younger groups.

Pattern Comparison	Weight	Height	BMI	Percentage Body Fat	Lean/Fat	Age	ECW/ICW
Combined Group	0.02	0.01	0.04	0.05	0.03	**0.51**	**0.32**
Older Group	<0.01	0.1	0.02	0.06	0.03	0.05	0.15
Younger Group	<0.01	<0.01	<0.01	<0.01	0.02	<0.01	<0.01
**Picture Sequence Memory**							
Combined Group	0.01	0.01	<0.01	<0.01	<0.01	**0.24**	0.11
Older Group	0.01	0.16	0.01	0.07	0.03	0.13	0.05
Younger Group	<0.01	0.02	0.02	0.01	0.02	<0.01	0.01
**Dimensional Change Card Sort**							
Combined Group	0.01	<0.01	0.02	0.05	0.05	**0.3**	**0.24**
Older Group	<0.01	0.06	0.01	0.02	<0.01	0.02	0.09
Younger Group	<0.01	<0.01	<0.01	0.01	<0.01	<0.01	0.01
**Flanker Inhibitory Control and Attention**							
Combined Group	0.01	0.03	0.04	0.12	0.11	**0.42**	**0.38**
Older Group	0.01	0.08	<0.01	0.01	<0.01	0.05	**0.24**
Younger Group	<0.01	0.02	<0.01	0.08	0.04	0.02	0.04
**List Sorting Working Memory**							
Combined Group	<0.01	<0.01	<0.01	0.01	<0.01	0.08	0.06
Older Group	0.04	0.02	0.03	<0.01	<0.01	0.02	0.04
Younger Group	<0.01	0.01	<0.01	<0.01	0.02	0.01	<0.01

**Table 3:** Depicts forward stepwise regression (F-to-enter: 1.6, F-to-remove: 1.5). Sex, percentage body fat, BMI, age, lean/fat, height, weight, and extracellular to intracellular ratio (ECW/ICW) were independent variables. Flanker score was the dependent variable.

Combined Group	Variable	R-Squared
Step 1	Age	0.42
Step 2	ECW/ICW	0.48
Young Group		
Step 1	Young Percentage Body Fat	0.08
Step 2	Young BMI	0.11
Step 3	Age	0.13
Step 4	Lean/Fat	0.16
Old Group		
Step 1	ECW/ICW	0.24
